# Stimulated whole salivary flow rate: The most appropriate technique for assessing salivary flow in Sjögren syndrome

**DOI:** 10.4317/medoral.24736

**Published:** 2021-03-27

**Authors:** Carlos Alvariño, Leticia Bagan, Judith Murillo-Cortes, Javier Calvo, Jose Bagan

**Affiliations:** 1 Assistant professor of Dentistry, Universidad Europea, Valencia, Spain; 2 Assistant professor of Oral Medicine, Valencia University, Spain; 3 Head Section of Stomatology and Maxillofacial Surgery. University General Hospital, Valencia; 4 Head service of Rheumatology. University General Hospital, Valencia, Spain; 5 Professor of Oral Medicine Valencia University. Head Service of Stomatology and Maxillofacial Surgery. University General Hospital, Valencia, Spain

## Abstract

**Background:**

We sought to determine the most appropriate method for measuring salivary flow to aid the diagnosis of Sjögren's syndrome (SS). Specifically, we compared the unstimulated whole salivary flow rate (UWSFR) with the stimulated whole salivary flow rate (SWSFR).

**Material and Methods:**

This case-control study comprised one group of 103 patients with SS and a control group of 50 healthy people. We measured the UWSFR and SWSFR in both groups according to the guidelines established by Navacet (1993).

**Results:**

The UWSFR and SWSFR were significantly lower in the patient group compared with the controls (*p* < 0.01). Among the participants in the patient group, we found a decreased UWSFR in 84 individuals (81.5%) and a decreased SWSFR in 90 individuals (87.4%). We encountered difficulties obtaining saliva in 37 (35.9%) patients during the UWSFR test, and in 12 (11.7%) patients during the SWSFR test. There was no significant statistical difference in the UWSFR or SWSFR between patients with primary and secondary SS.

**Conclusions:**

Compared with the UWSFR, the SWSFR is a more suiTable and effective method for measuring salivary flow in patients with SS, as well as for qualitative analysis of the obtained saliva.

** Key words:**Sjögren syndrome, xerostomia, salivary glands.

## Introduction

Sjögren's Syndrome (SS) is a systemic autoimmune disease with an unknown etiology. It is characterized by the infiltration of T lymphocytes (LT) and B lymphocytes (LB) into exocrine glands, mainly the salivary and lacrimal glands. The resulting progressive destruction of the glands can decrease their excretory capacity, giving rise to sicca complex or dry eye syndrome, which is characterized by the sensation of a dry mouth (xerostomia) and dry eyes (xerophthalmia).

In the literature, the American-European Consensus Criteria (AECC), which include two subjective and four objective criteria, have been used most frequently in the diagnosis of SS (1). A patient can be diagnosed with SS if they meet four of the six criteria or at least three of the objective criteria. In 2016, the American College of Rheumatology (ACR) and European League Against Rheumatism (EULAR) published new criteria for diagnosing SS. The ACR-EULAR criteria (2) do not include the subjective criteria from previous guidelines. Instead, they give greater weight to quantifiable measures, such as serum anti-SSa antibodies and lymphocytic foci in glandular histological samples. A score above 4 in this diagnostic system is required for a diagnosis of SS.

In both of the above-mentioned classification systems, unstimulated whole salivary flow (UWSFR) is considered to be a valid diagnostic criterion. However, many authors have pointed out that the UWSFR is highly influenced by other factors (e.g., age, circadian cycles, room temperature, medication, sample collection technique, diseases, etc.), and have proposed that the stimulated saliva (SWSFR) test be used as a more reliable way of evaluating glandular function in patients suspected of having SS (3).

The main objective of our study was to compare the utility of the two salivary measurement techniques, i.e., the UWSFR and SWSFR, for diagnosing SS.

## Material and Methods

Between 2016 and 2020, we assessed 103 patients diagnosed with SS. The patients were initially admitted to the Rheumatology Department at the General University Hospital of Valencia, and then referred to the Stomatology Maxillofacial Surgery Service for analysis of saliva flow rate and salivary gland biopsies. The mean age of the participants was 62.16 ± 11.76 years, and there were 97 (94.2%) women and 6 (5.8%) men. In terms of SS type, 76 patients had primary SS and 27 had secondary SS. The inclusion criteria for the SS group (103 participants in total) were consistent with the diagnostic criteria established by both the AECG [1] and ACR-EULAR [2]. We excluded all patients with a history of chemotherapy and or radiotherapy of the head and neck, as well as those undergoing treatment with parasympathomimetic drugs such as pilocarpine or cevimeline.

We recruited a control group comprised of 50 healthy persons. The two groups were matched in terms of age and gender. The control group included no participants with rheumatological diseases, symptoms of oral dryness, or a history of chemotherapy and or radiotherapy of the head and neck, and no individuals who were undergoing treatment with drugs that could affect salivary secretion.

For all participants, we measured the unstimulated salivary flow rate and stimulated salivary flow rate using the technique recommended by Navazesh (4).

The cut-off value for a diagnosis of hyposalivation was a flow rate ≤ 0.1 ml/min over 5 minutes (< 0.5 ml collected in total) for the UWSFR, and < 0.7 ml/min over 5 minutes (< 3.5 m collected in total l) for the SWSFR, according to previous studies (5).

As descriptive statistics, we calculated mean, minimum, maximum and standard deviation values for the quantitative variables. Using the chi-square test, we assessed the associations between the qualitative variables. Finally, we analyzed the correlation between the UWSFR and SWSFR using Pearson’s correlation coefficient. We set the significance level at *p* < 0.05 for all analyses.

## Results

The UWSFR and SWSFR values are given in Table 1. Compared with the control group, the SS group exhibited a lower UWSFR and SWSFR (*p* < 0.01). The UWSFR was decreased in 84 cases (81.5%) in the SS group, while the SWSFR was decreased in 90 cases (87.4%). We were unable to obtain any saliva in 37 (35.9%) of the SS patients using the UWSFR test. With the SWSFR test, there were only 12 (11.7%) participants for whom we did not obtain any saliva. Within the SS group, the Pearson correlation coefficient between the UWSFR and SWSFR was significant (r = 0.57, *p* < 0.01).

There was no statistical difference between the 76 patients with primary SS and the 27 with secondary SS in terms of the UWSFR or SWSFR (Table 1).

## Discussion

As we described in the Material and Methods, our group of 103 subjects with SS included both primary and secondary SS patients. This is in contrast to the vast majority of studies that only included SS patients who had no comorbid autoimmune diseases.

As shown in Table 2, most previous studies that measured the UWSFR reported a mean flow rate of 0.04–0.09 ml/min (6-12); this is in agreement with the mean rate in our SS patients. Regarding the SWSFR, studies that quantitatively measured this variable reported a mean flow of 0.4–0.6 ml/min, which is very similar to that in our 103 subjects with SS.

**Table 1:**
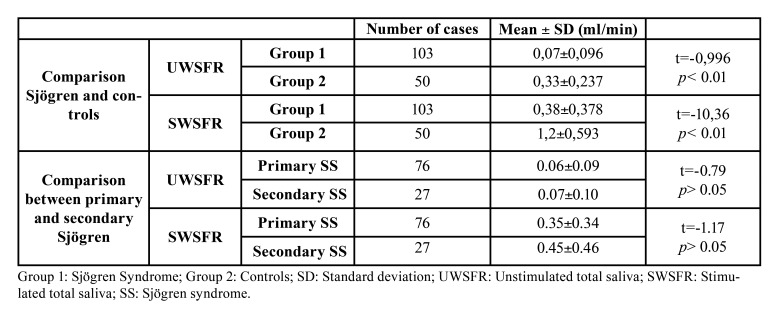
UWSFR and SWSFR values in SS patients and controls.

**Table 2:**
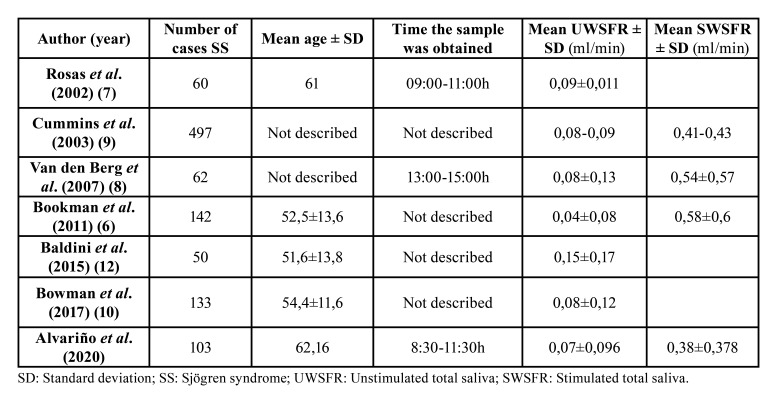
Comparison of UWSFR and SWSFR values in SS patients among studies that included more than 50 cases.

Sialometry data can be influenced by many factors, such as the age of the patient (13,14), the time at which the test is performed (saliva production changes according to circadian rhythms), medication, and the technique used to collect saliva (15). We considered all of these factors when performing sialometry in the present study. Variations in such factors could explain the inconsistencies among previous reports, which found decreases in salivary secretion ranging from 34–91% in SS patients (3,11,16,17).

When we compared the present results to previous findings, we found the following. In the Sjögren Big Data Project, which comprised a large international cohort of SS patients, Brito-Zerón *et al*. [2018] reported that the percentage of patients with a lower saliva rate at rest was 75.2% (16). This was slightly lower than the proportion in the present study. Furthermore, among SS patients in a large international cohort study, Shiboski *et al*. (11) found that a lower percentage, i.e., 62%, had altered sialometry. Another study reported that only 34.6% of patients with SS had altered sialometry (3). However, Park *et al*. reported a higher proportion of SS patients with decreased sialometry (18), i.e., 91%.

Regarding the decrease in stimulated salivary flow rate, 61.8% of the 142 patients of Bookman *et al*. (6) had altered stimulated sialometry; this was considerably lower than the proportion in the present study. This discrepancy can probably be explained by the different criterion used by Bookman *et al*. (6); they considered stimulated sialometry to be abnormal when the SWSFR was < 0.6 ml/min, while we used a cut-off value of 0.7 ml/min. Furthermore, the salivary collection method used by Bookman *et al*. (6) was different from ours. Specifically, while they were limited to collecting stimulated saliva for only 1 minute, we collected saliva for 5 minutes.

To the best of our knowledge, the only other study to measure both the unstimulated and stimulated salivary flow rate in SS patients was that of Serrano *et al*. [2020] (19). Their analysis showed that 60.7% of SS patients had a decreased UWSFR. This percentage is considerably lower than that in our study, but we are unable to explain this difference.

We would like to emphasize that we did not find any statistical difference in the unstimulated or stimulated salivary flow rate between the patients with primary and secondary SS. These findings are consistent with the proposal, made by a number of researchers, that distinguishing between primary and secondary SS is not necessary. Indeed, there are no significant differences in the main disease characteristics between subjects with any form of SS and those with related autoimmune disorders.

In conclusion, we consider it remarkable that among our 103 subjects with SS, abnormal SWSFR results were more frequent than abnormal UWSFR results. Currently, the diagnostic criteria for SS (1,2) consider only the UWSFR. When using the UWSFR test, we were not able to obtain saliva in 35.9% of our participants with SS. However, this proportion fell to 11.7% when we used the SWSFR. This result, together with previous data showing that the UWSFR at rest is more influenced than the SWSFR by external factors, indicates that stimulated sialometry is superior to the UWSFR as a diagnostic marker for SS. Also, the larger quantity of saliva collected in the SWSFR test can facilitate other analytical processes (20), such as examination of methylome signatures (hypermethylated genes), transcriptome signatures (miRNA), microbiome signatures, and proteomic signatures. These procedures are more challenging when smaller quantities of saliva are available, which is a drawback of the UWSFR test.
